# Oxygen consumption and acid secretion in isolated gas gland cells of the European eel *Anguilla anguilla*

**DOI:** 10.1007/s00360-022-01432-x

**Published:** 2022-03-14

**Authors:** Victoria Drechsel, Gabriel Schneebauer, Adolf M. Sandbichler, Birgit Fiechtner, Bernd Pelster

**Affiliations:** 1 Institut für Zoologie, Leopold-Franzens-Universität Innsbruck, Technikerstr.25, 6020 Innsbruck, Austria; 2 Center for Molecular Biosciences, University of Innsbruck, Innsbruck, Austria; 3 Animal Physiological Ecology, Institute of Evolution and Ecology, University of Tübingen, Tübingen, Germany; 4 Institute for Human Genetics, Medical University Innsbruck, Innsbruck, Austria

**Keywords:** Swimbladder, Metabolism, Oxygen consumption, European eel, Lactate

## Abstract

Swimbladder gas gland cells are known to produce lactic acid required for the acidification of swimbladder blood and decreasing the oxygen carrying capacity of swimbladder blood, i.e., the onset of the Root effect. Gas gland cells have also been shown to metabolize glucose via the pentose phosphate shunt, but the role of the pentose phosphate shunt for acid secretion has not yet been evaluated. Similarly, aerobic metabolism of gas gland cells has been largely neglected so far. In the present study, we therefore simultaneously assessed the role of glycolysis and of the pentose phosphate shunt for acid secretion and recorded oxygen consumption of isolated swimbladder gas gland cells of the European eel. Presence of glucose was essential for acid secretion, and at glucose concentrations of about 1.5 mmol l^−1^ acid secretion of gas gland cells reached a maximum, indicating that glucose concentrations in swimbladder blood should not be limiting acid production and secretion under physiological conditions. The data revealed that most of the acid was produced in the glycolytic pathway, but a significant fraction was also contributed by the pentose phosphate shunt. Addition of glucose to gas gland cells incubated in a glucose-free medium resulted in a reduction of oxygen uptake. Inhibition of mitochondrial respiration significantly reduced oxygen consumption, but a fraction of mitochondria-independent respiration remained in presence of rotenone and antimycin A. In the presence of glucose, application of either iodo-acetate inhibiting glycolysis or 6-AN inhibiting the pentose phosphate shunt did not significantly affect oxygen uptake, indicating an independent regulation of oxidative phosphorylation and of acid production. Inhibition of the muscarinic acetylcholine receptor caused a slight elevation in acid secretion, while forskolin caused a concentration-dependent reduction in acid secretion, indicating muscarinic and c-AMP-dependent control of acid secretion in gas gland cells.

## Introduction

The energy metabolism of swimbladder gas gland cells of physoclist fish is peculiar in that they typically experience hyperoxic conditions, but glucose is mainly converted to lactic acid to generate sufficient protons for the acidification of swimbladder blood (D’Aoust 1970; Deck 1970; Pelster 1995b; Pelster and Scheid 1993). Lactic acid production and secretion are considered the main sources of swimbladder blood acidification. On acidification, hemoglobin releases oxygen (Root effect), which is essential for the generation of hyperoxic oxygen partial pressures (Pelster and Randall 1998; Pelster and Weber 1991; Pelster 2001, 2021) required to fill the swimbladder with oxygen, even under conditions of elevated hydrostatic pressure. The initial increase in oxygen partial pressure in a second step is multiplied by countercurrent concentration in the rete mirabile of the swimbladder, so that hyperbaric oxygen partial pressures can be generated, sufficient to explain the secretion of oxygen against hydrostatic pressures of several 10 s or even more than 100 atmospheres (Kuhn et al. 1963; Pelster 2001).

Gas gland cells of the American eel and of the gulf toadfish have been shown to produce CO_2_ in the pentose phosphate shunt (Pelster et al. 1994; Walsh and Milligan 1993). The CO_2_ diffuses along the partial pressure gradient into the swimbladder lumen, contributing to the filling of the swimbladder, but also into the blood, supporting the acidification of the blood. The possible contribution and the importance of the pentose phosphate shunt to acid secretion of gas gland cells and acidification of the blood, however, have never been addressed. In addition, NADPH is produced in the pentose phosphate shunt, which may affect the redox equilibrium in the cell.

Analysis of a number of different fish species revealed that mitochondria are not numerous in gas gland cells (Dorn 1961; Copeland 1969; Jasinski and Kilarski 1969; Morris and Albright 1975), but enzyme activities of the aerobic metabolism (Ewart and Driedzic 1990; Pelster and Scheid 1991; Walsh and Milligan 1993) as well as oxygen uptake have been reported. Oxygen uptake of a saline perfused swimbladder preparation of the European eel, which did not secrete any gas into the swimbladder, was measured, and it was significantly lower than the oxygen uptake of blood perfused eel swimbladder tissue secreting gas into the swimbladder lumen. The rate of gas secretion was correlated to the rate of lactate formation, but the elevated oxygen uptake indicated that aerobic metabolism is also of importance in the active swimbladder (Pelster 1995b; Pelster and Scheid 1992). The role of aerobic metabolism, however, has not been carefully analyzed.

Regulation of gas gland cell metabolism, a crucial parameter influencing the rate of gas secretion (Pelster and Scheid 1992), remains enigmatic. Cholinergic nerve endings have been detected in swimbladder tissue of cod (McLean and Nilsson 1981; Nilsson 1983). Acetylcholine, respective the cholinomimetic drug carbachol, have been reported to stimulate lactate production in cod, but the effect was not pronounced and particularly low at acidic pH of 6.5, which is to be expected in active gas gland cells (Ewart and Driedzic 1990; Pelster 1995a). Activation of adenylate cyclase in turn has been shown to reduce the rate of acid secretion in primary cultured eel gas gland cells (Pelster and Pott 1996). An elevation of acid secretion, however, could not be elicited in these experiments.

Aim of the present study therefore was to analyze oxygen consumption of isolated gas gland cells of the European eel and to assess the importance of lactate formation and of the pentose phosphate shunt for acid secretion of the cells.

## Materials and methods

### Animals

European eels *Anguilla anguilla* were caught by local fishermen in Lake Constance, Bregenz, Austria, and kept in an outdoor freshwater basin at the Institute of Zoology at the University of Innsbruck. Two days prior to sampling, fish were transferred into an indoor freshwater aquarium. Morphometrics and the silvering stage of eels used for the study are summarized in Table 1.

**Table 1:** Morphometrics of European eel *Anguilla anguilla* used for the experiments (mean ± SD, *N* = 59)

	Mean ± SD
Body mass [g]	628 ± 298
Body length [cm]	76 ± 11
Pectoral fin length [mm]	32.8 ± 5.8
Horizontal eye diameter [mm]	7.96 ± 1.41
Vertical eye diameter [mm]	7.54 ± 1.27
Silver Index	2.39 ± 0.71
Ocular Index	6.24 ± 1.49

The silvering index was calculated according to (Durif et al. 2005) and ocular index according to (Pankhurst 1982)

### Preparation of gas gland cells

Eels were anesthetized with 2-phenoxyethanol (1 ml l^−1^), and subsequently de-cerebrated and spinally pithed. The abdominal wall was opened ventrally, the swimbladder carefully dissected and cleaned of connective tissue. Procedures for primary culture of European eel gas gland cells have been published previously by Pelster and Pott (1996) and were slightly modified for the current study. Briefly, the tissue was washed three times with ice-cold Hank’s buffered salt solution without Ca^2+^ and Mg^2+^ (HBSS, Thermo Fisher Scientific, Germany; pH 7.4), chopped into smaller pieces and then washed again in ice-cold HBSS. Tissue pieces were digested with trypsin–EDTA (0.25% EDTA, Thermo Fisher Scientific, Germany) in HBSS (1:5) for 30 min on a shaker at 200 rpm at room temperature, and this procedure was repeated four times. After the first 30 min, the resulting cell solution was discarded as it contained primarily erythrocytes and fibroblasts. After the second, third and fourth time, the solution was filtered through a 70 µm cell strainer and gas gland cells as well as cell clusters dissolved from the tissue were transferred to ice-cold stopping solutions (1:10 fetal bovine serum in HBSS 1.26 mM CaCl_2_, 0.49 mM MgCl_2_ and 0.41 mM MgSO_4_, Thermo Fisher Scientific, Germany). The three cell suspensions were centrifuged for 5 min at 800 rpm and 4 °C. The cell pellets were re-suspended in culture medium (Leibovitz’s L-15 Medium supplemented with 15% fetal bovine serum, 1% Penstrep, 0.12% Gentamycin, Insulin-Transferrin-Selenium-A 5 µg ml^−1^, EGF 0.02 µg ml^−1^ (all Thermo Fisher Scientific, Germany), L-glutamine 2 mmol l^−1^, putrescine 16 µg ml^−1^, pituitary gland extract 0.5 µg ml^−1^, and progesterone 6.29 ng ml^−1^ (all Sigma-Aldrich, Germany)).

Isolated gas gland cells were seeded into collagen-S (Sigma-Aldrich, Germany) coated culture dishes and incubated at 22 °C. After 48 h, the cultures were washed with HBSS containing Ca^2+^ and Mg^2+^ and the culture medium was replaced every second day until reaching confluency in at least one of the culture dishes, usually 5–7 days after seeding.

### Seahorse XFp extracellular flux analyzer measurements

Oxygen consumption rate (OCR) and extracellular acidification rate (ECAR) of gas gland cells were measured with a Seahorse XFp Analyzer (Agilent, USA) according to the manufacturer’s instructions. Cells were re-seeded into Seahorse XFp cell culture mini-plates (Agilent, USA) at a density of 15,000 cells per well and cultured for two more days. 60 min prior to the measurements, the medium in the mini-plates was changed to XF Base Medium Minimal DMEM containing 2 mmol l^−1^ l-glutamine, adjusted to pH 7.4. To assess the relation between the rate of available glucose concentration and the rate of acid secretion and OCR, basal metabolic rate was determined for 36 min before the glucose concentration in the medium was stepwise (0.25 mM steps) increased to 3.75 mmol l^−1^ and OCR and ECAR were measured for about 50–60 min for each step.

To assess the role of the different metabolic pathways, again baseline metabolic rate (basal level of acidification and respiration in the absence of glucose) of the cells was measured prior to the addition of various compounds, and the influence of these compounds on OCR and ECAR was recorded over a period of 30–60 min. Iodoacetic acid (100 µmol l^−1^; IAA) was used to inhibit the glycolytic pathway enzyme Glyceraldehyde-3-phosphate dehydrogenase, and 6 amino-nicotinamide (5 µmol l^−1^; 6-AN) was applied for inhibition of Glucose-6-phosphate dehydrogenase, a key enzyme of the pentose phosphate shunt. Cells were incubated with or without 6-AN 12 h prior to the injection of a saturating concentration of glucose (10 mmol l^−1^). Rotenone (0.5 µmol l^−1^; inhibiting complex I) and antimycin A (AMA; 0.5 µmol l^−1^; inhibiting complex III) were used to inhibit mitochondrial electron transport.

To assess the importance of intracellular signaling pathways for the control of metabolic activity of gas gland cells, various compounds were selected as agonists or antagonists in specific messenger pathways. In these experiments, glucose concentration was set to 4 mmol l^−1^ and OCR and ECAR were recorded for 30 min. All chemicals were purchased from Sigma-Aldrich, Vienna, Austria (https://www.sigmaaldrich.com/life-science.html), and are listed with their putative effect and used concentration in Table 2. All experiments were performed at the culturing temperature of 22 °C.

**Table 2:** List of chemicals that act as agonists or antagonists in specific messenger pathways or in parts of the glucose metabolism

Chemical (Abbreviation)	Function	*N*	Final concentration
Rotenone (Ro)	Inhibits mitochondrial respiration by inhibiting Complex I of the electron transport chain		0.5 µM
Antimycin A (AMA)	Inhibits mitochondrial respiration by inhibiting Complex III of the electron transport chain		0.5 µM
6-Aminonicotinamid (6-AN)	Inhibits glucose-6-phosphate dehydrogenase, i.e., inhibits the pentose phosphate pathway		5 µM–1 mM
Iodoacetate (IAA)	Inhibits glyceraldehd-3-phosphate-dehydrogenase (GAPDH), i.e., inhibits glycolysis		100 µM
2-Deoxy-d-glucose (2-DG)	Inhibits glucose metabolism through competitive binding to hexokinase		50 mM
Acetylcholine chloride	Neurotransmitter	5	10^–4^, 10^–5^, 10^–6^, 10^–7^ M
Hexamethonium chloride	Inhibitor of nicotinic acetylcholine receptors	5	10^–5^, 10^–6^, 10^–7^, 10^–8^ M
Dicyclomine hydrochloride	Competitive muscarinic acetylcholine receptor antagonist	5	10^–5^, 10^–6^, 10^–7^, 10^–8^ M
Forskolin	Adenylyl cyclase agonist	5	10^–5^ M, 10^–7^ M
Dopamine-hydrochloride	Neurotransmitter	4	10^–5^ M, 10^–7^ M
γ-Aminobutyric acid (GABA)	Neurotransmitter	5	10^–5^ M, 10^–7^ M
Serotonin hydrochloride	Neurotransmitter	3	10^–6^ M, 10^–7^ M

They were all dissolved in XF Base Medium Minimal DMEM pH 7.4 and used in the given final concentrations

### Measurement of NADPH/NADP^+^ concentration

Cellular NADPH/NADP^+^ ratio was quantified in white 96-well plates (Sarstedt, Germany) using the NADP^+^/NADPH Glo™ Assay kit (Promega, USA). Levels of cellular NADP^+^ or NADPH could individually be measured after treatment with acid or base conditions, respectively. For this, cells in 50 µl PBS were lysed with the same volume of base solution containing 1% DTAB. To measure NADP^+^, 50 µl of the lysed cells was transferred into a new well and 25 µl of 0.4 N HCl was added (acid treatment). After 15 min at 60 °C, the plate was incubated for 10 min at room temperature before the acid-treated NADP^+^ wells received another 25 µl of Trizma base, and the base-treated NADPH wells received 50 µl of HCl/Trizma solution. After reconstituting, the NADP^+^/NADPH Glo Detection reagent according to the manufacturers protocol 100 µl of the reagent was added 1:1 to each well holding acid or base-treated cells. After a brief shake, cells were left for 30 min at room temperature prior to recording luminescence (1 s integration time) in a Multimode plate reader (Enspire, Perkin Elmer).

### Data analysis and statistics

The Seahorse Wave software (Version 2.4, Agilent) was used to design, run, report, and export the files from the Seahorse instrument. Measurements of each cell preparation were performed in triplicate, N gives the number of cell preparations. Measurement values were normalized to the maximum ECAR and OCR reached after supplying the saturating concentration of glucose (10 mmol l^−1^ or 4 mmol l^−1^ glucose, respectively), which was then defined as 100% acidification rate, or 100% oxygen uptake rate. In the ascending glucose concentration experiment, the point where acidification and OCR reached a plateau was set to 100%. All additional measurement points were calculated accordingly.

Statistical analysis was performed in Sigma Plot (Version 14.0, Systat Software). Comparison between treatments was performed using paired Student’s *t *test or, when comparing more than two groups, one-way ANOVA followed by all-pairwise multiple comparison procedures (Dunn’s method, Tukey’s Test). Significance was accepted for *p* < 0.05.

## Results

**Fig. 1:**
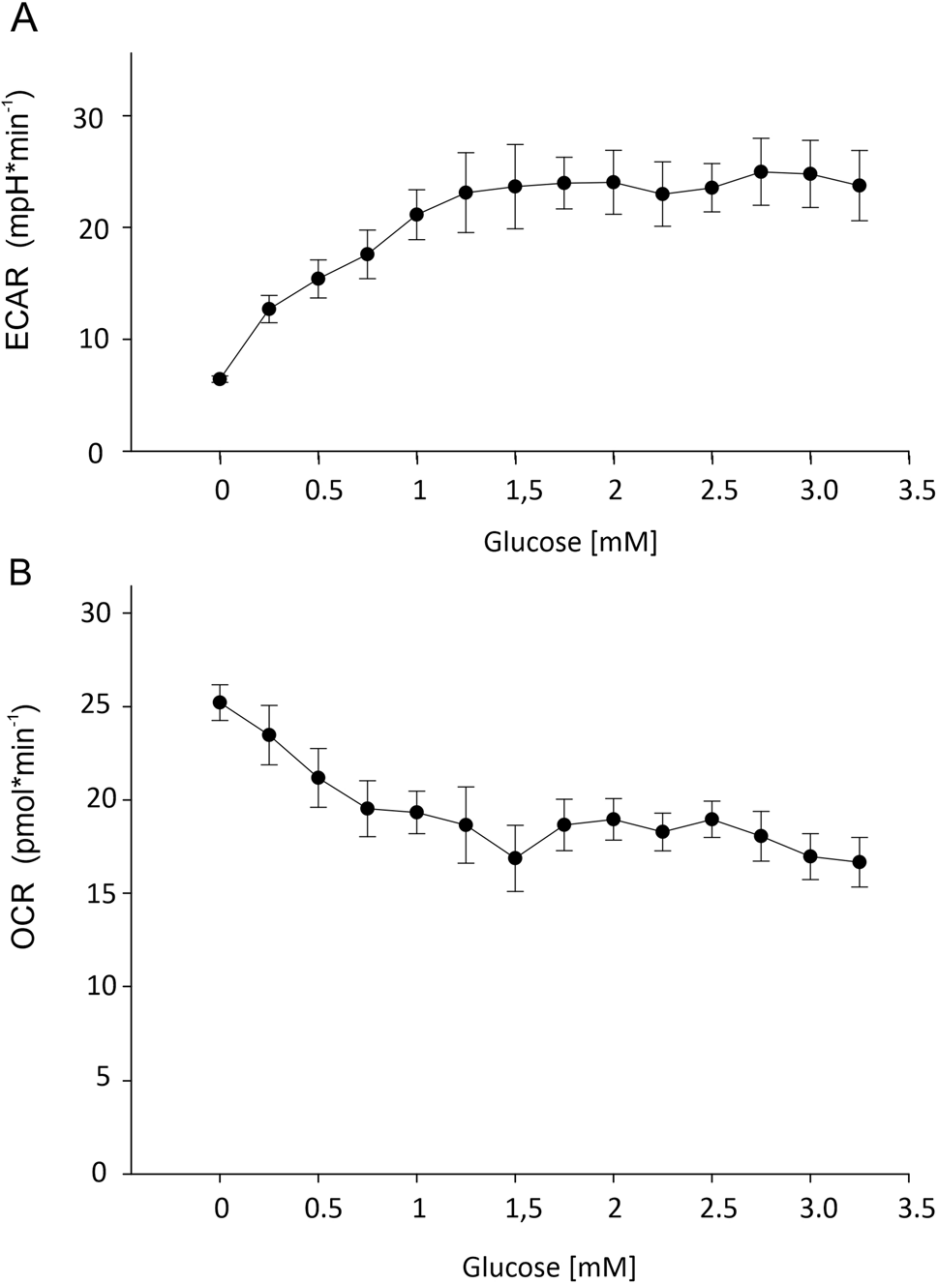
Acid secretion (ECAR) (**A**) and oxygen uptake (OCR) (**B**) in relation to increasing glucose concentrations. ECAR rapidly increased with increasing glucose concentrations and leveled off at glucose concentrations above 1.5 mmol l^−1^. OCR decreased with increasing glucose concentrations and remained stable at concentrations above 1.5 mmol l^−1^; *N* = 6

A stepwise increase in glucose concentration caused a stepwise increase in the rate of acid secretion, reaching a maximum at a glucose concentration of only 1.5 mmol l^−1^ (Fig. 1A). Oxygen consumption slowly decreased with increasing glucose concentration and remained constant at glucose concentrations above 1–1.5 mmol l^−1^ (Fig. 1B).

**Fig. 2:**
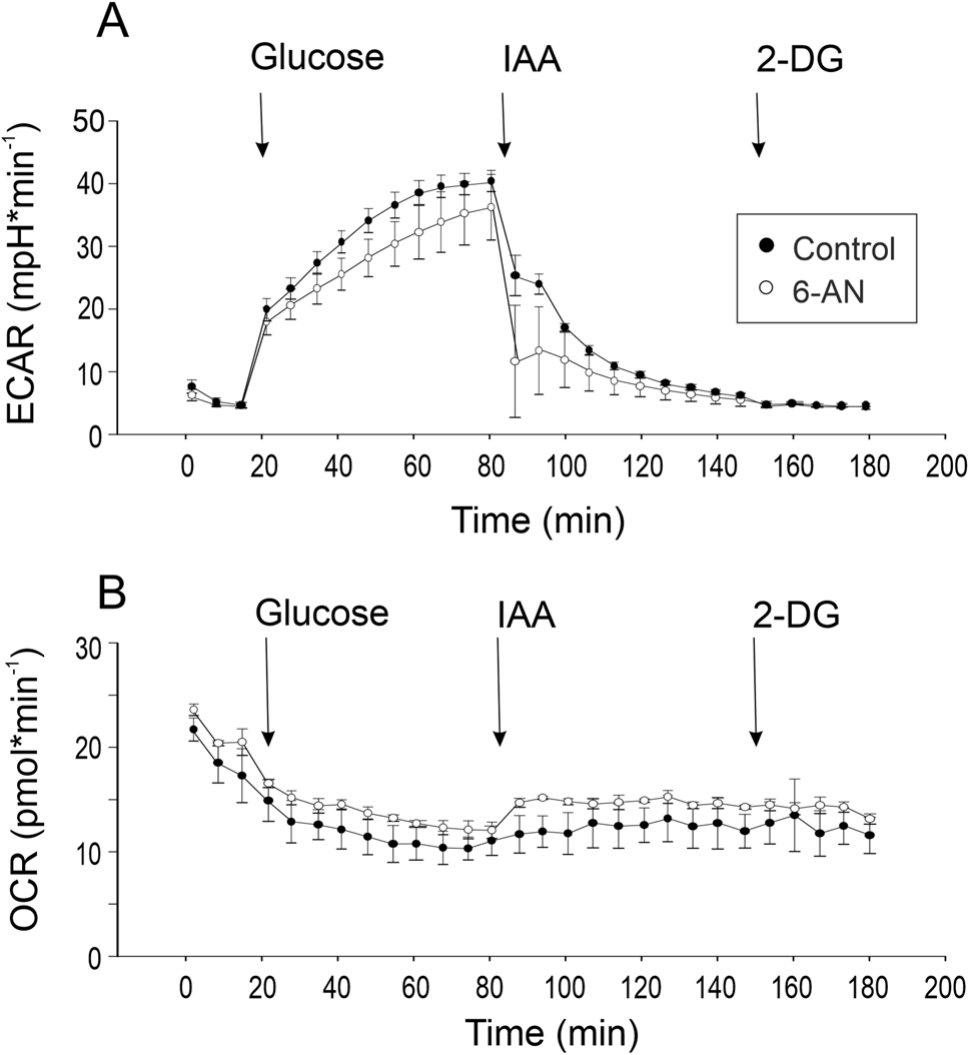
Time course of a typical experiment testing the effect of an inhibition of glycolysis by application of IAA (100 µM) to swimbladder gas gland cells either pre-incubated with 6-AN or to control gas gland cells on acid secretion (ECAR) (**A**) and on oxygen consumption (OCR) (**B**). The figure shows triplicate measurements of a single preparation

As a next step, we assessed the role of lactate production and the pentose phosphate shunt for acid secretion and oxygen uptake in gas gland cells pre-incubated with 6-AN to inhibit the pentose phosphate shunt and in control cells. Figure 2 shows a typical time course of these experiments. Cells initially were incubated without glucose. Addition of a saturating concentration of glucose (10 mmol l^−1^) resulted in a rapid increase in acid secretion, ECAR increased from about 5 mpH min^−1^ to between 30 and 40 mpH min^−1^ in 6-AN pre-incubated cells as well as in control cells (Fig. 2A). Addition of iodo-acetate (IAA) to inhibit glycolysis caused a rapid decrease in ECAR down to the initial values in both groups, and addition of 2-deoxyglucose (2-DG) to completely block glucose metabolism resulted in another slight decrease in ECAR (Fig. 2A). Oxygen uptake decreased in 6-AN pre-incubated cells as well as in control cells after addition of glucose (Fig. 2B). Inhibition of glycolysis by addition of IAA in this experiment appeared to slightly increase oxygen uptake, but this effect was not significant. In both groups, addition of 2-DG had no effect on oxygen uptake. For quantitative analyses, values of the last three data points of a specific treatment were averaged.

**Fig. 3:**
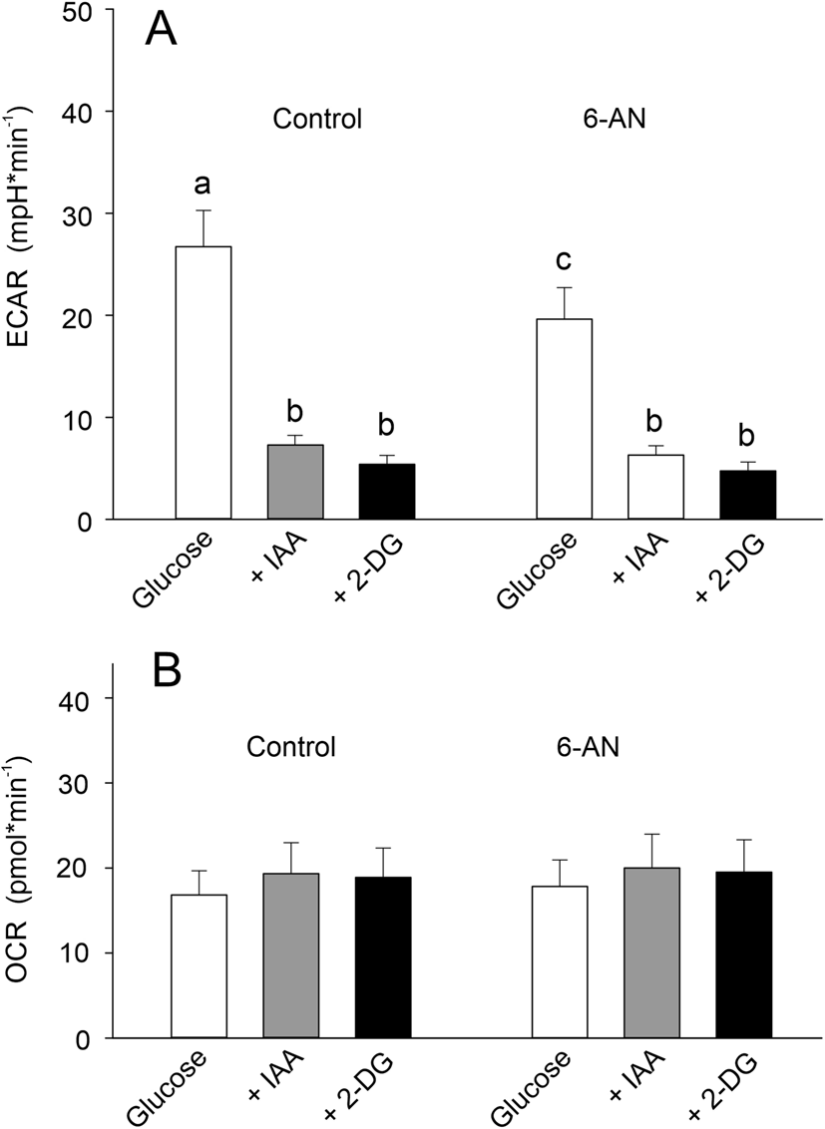
**A** ECAR in the presence of glucose and after inhibition of the glycolytic pathway by application of iodo-acetate (IAA; 100 µmol l^−1^) and after addition of 2-deoxyglucose (50 mmol l^−1^) with (6-AN; 5 µmol l^−1^) and without inhibition (control) of the pentose phosphate shunt using 6-AN. In the presence of glucose, ECAR was significantly lower in the presence of 6-AN. **B** OCR recorded under these conditions was not significantly affected following inhibition of the glycolytic pathway. Different small letters indicate differences between treatments; *N* = 14; *p* < 0.05

Quantitative analysis of these experiments revealed that acid secretion was reduced to 25% of the control value by addition of IAA (Fig. 3A). Addition of 2-deoxyglucose caused an additional 7% reduction in acid secretion (Fig. 3A). In cells pre-incubated with 6-AN, the rate of acid secretion was significantly lower than in control cells (Fig. 3A). Addition of IAA to these cells reduced acid secretion to 32% of the control value, and addition of 2-DG caused an additional reduction in ECAR, but in controls and also in the presence of 6-AN, ECAR rates determined after completely blocking glucose metabolism using 2-DG, were not significantly different from ECAR rates determined in the presence of IAA (Fig. 3A). Inhibition of the glycolytic pathway using IAA had no effect on oxygen uptake of gas gland cells, neither in the presence nor in the absence of 6-AN (Fig. 3B).

**Fig. 4:**
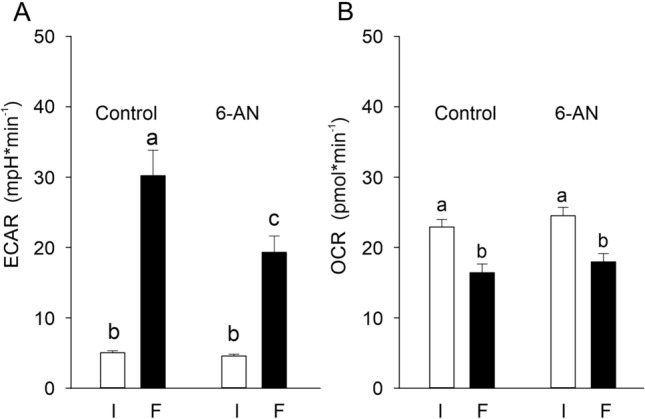
Acid secretion rate (ECAR, in mpH min^−1^) (**A**) and oxygen consumption (OCR, in pmol min^−1^) (**B**) of cultured primary gas gland cells in the absence (initial; I) and after addition of glucose (final; F) with and without inhibition of the pentose phosphate shunt using 6-AN (5 µmol l^−1^). Presence of 6-AN hardly affected OCR, but the increase in ECAR induced by addition of glucose was significantly diminished in the presence of 6-AN. Different small letters indicate differences between treatments; *N* = 14; *p* < 0.05

To more specifically assess the contribution of the pentose phosphate shunt to acid secretion, cells were incubated with 6-AN, to inhibit Glucose-6-phosphate dehydrogenase, for 12 h. Addition of glucose (10 mmol l^−1^) stimulated acid secretion resulting in a pH change from 5.0 ± 0.3 mpH min^−1^ to 30.2 ± 3.6 mpH min^−1^ in control cells (Fig. 4A). In the presence of 6-AN, the increase in acid secretion was significantly lower, raising pH from 4.5 ± 0.3 mpH min^−1^ to 19.3 ± 2.3 mpH min^−1^ (Fig. 4A). Oxygen uptake decreased by about 28% in response to the addition of a saturating concentration of glucose in control cells, a similar value (27%) was observed in cells incubated with 6-AN (Fig. 4B).

**Fig. 5:**
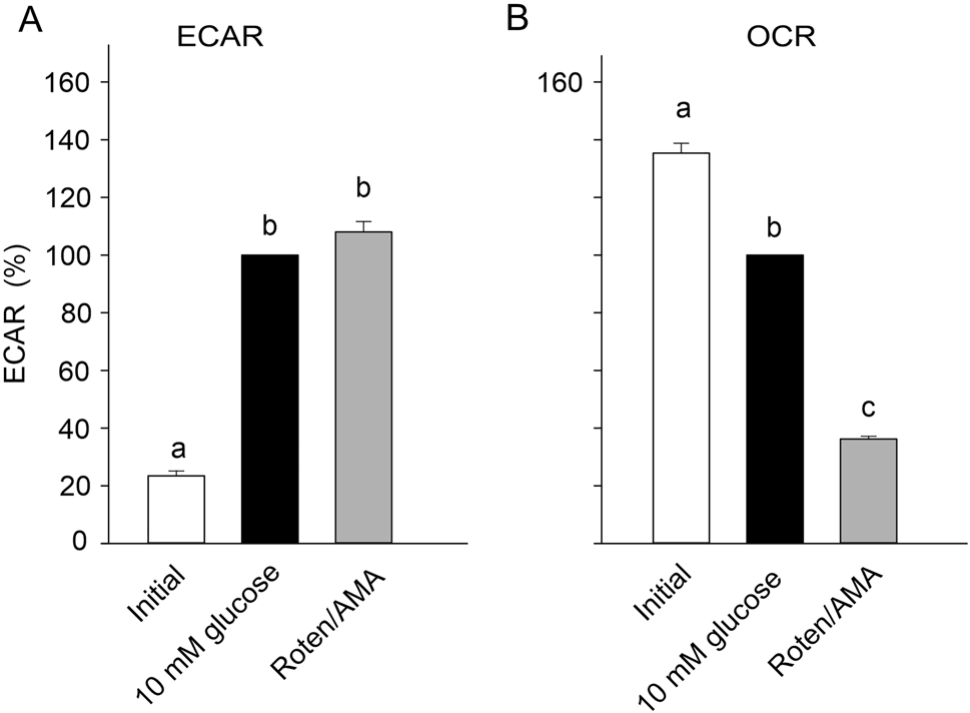
Acid secretion rate (ECAR) (**A**) and oxygen consumption (OCR) (**B**) of cultured primary gas gland cells in the absence of glucose (initial), in presence of 10 mM glucose and with inhibition of the respiratory chain (addition of rotenone, 0.5 µmol l^−1^ + antimycin A, 0.5 µmol l^−1^; Roten/AMA). OCR and ECAR recorded in presence of 10 mM glucose were set to 100%. Addition of glucose significantly reduced OCR and increased ECAR, inhibition of the respiratory chain significantly reduced OCR, but did not increase ECAR. Different small letters indicate differences between treatments; *N* = 14; *p* < 0.05

To quantify the role of glucose and of the aerobic metabolism, gas gland cells were initially incubated without glucose, followed by the addition of 10 mmol l^−1^ glucose. Addition of glucose resulted in a more than fourfold increase in acid secretion, and the addition of rotenone/antimycin A hardly affected acid secretion (Fig. 5A). Addition of glucose resulted in a 35% decrease in oxygen consumption, as observed in previous experiments. Addition of rotenone/antimycin A to evaluate the role of aerobic metabolism reduced oxygen uptake by more than 60% (Fig. 5B).

**Fig. 6:**
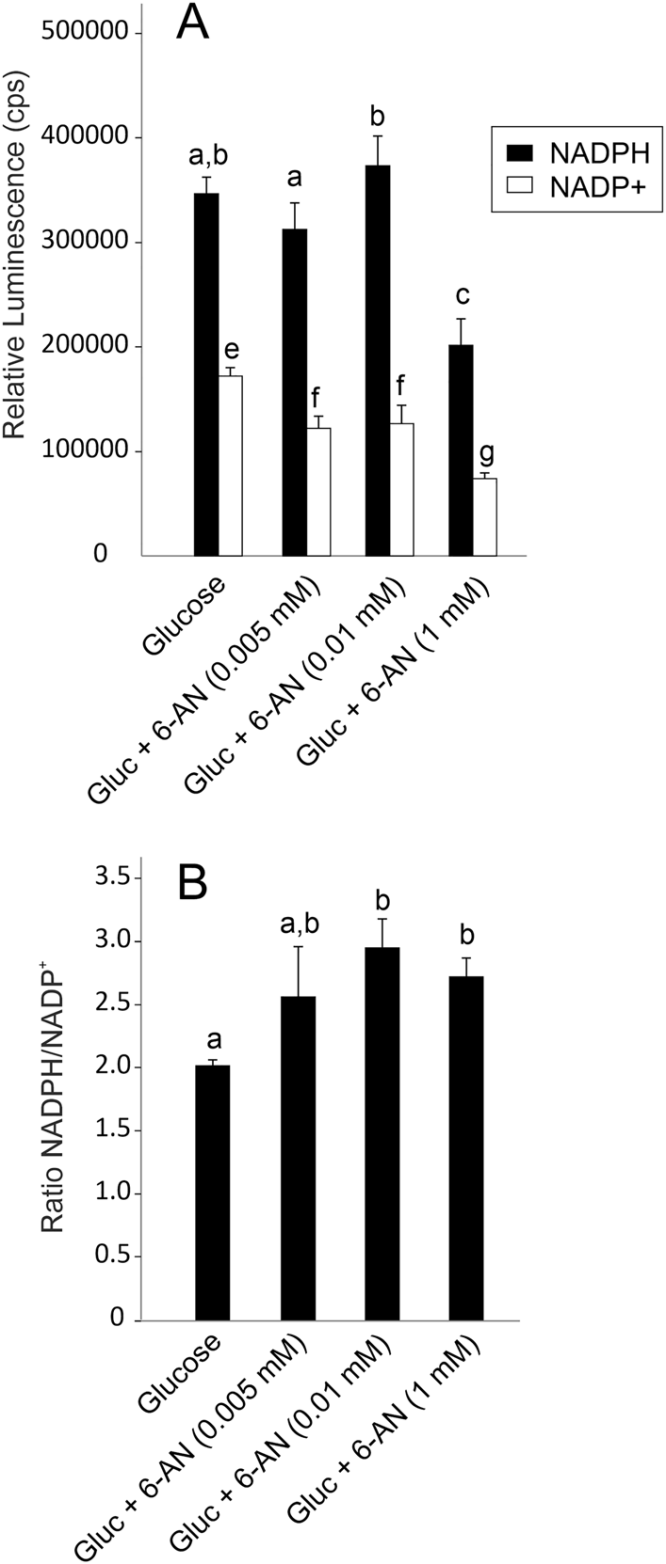
**A** The effect of inhibition of the pentose phosphate shunt by application of 6-AN on the concentration of the reduction equivalents NADPH and NADP^+^ (presented as Relative Luminescence, recorded in counts per second (cps)). Higher concentrations of 6-AN resulted in a decreased concentration of NADPH and NADP^+^ (**A**). The ratio of NADPH over NADP^+^ was significantly affected at higher concentrations of 6-AN (**B**). Different small letters indicate differences between treatments; *N* = 3; *p* < 0.05

There was some variation in the concentration of NADPH in the presence of 6-AN, but at the highest concentration of 6-AN, it was significantly decreased. The concentration of NADP^+^ decreased with increasing concentrations of 6-AN (Fig. 6A). Accordingly, the ratio of NADPH over NADP^+^ increased and was significantly elevated at 0.01 mmol l^−1^ and 1.0 mmol l^−1^ 6-AN (Fig. 6B).

**Fig. 7:**
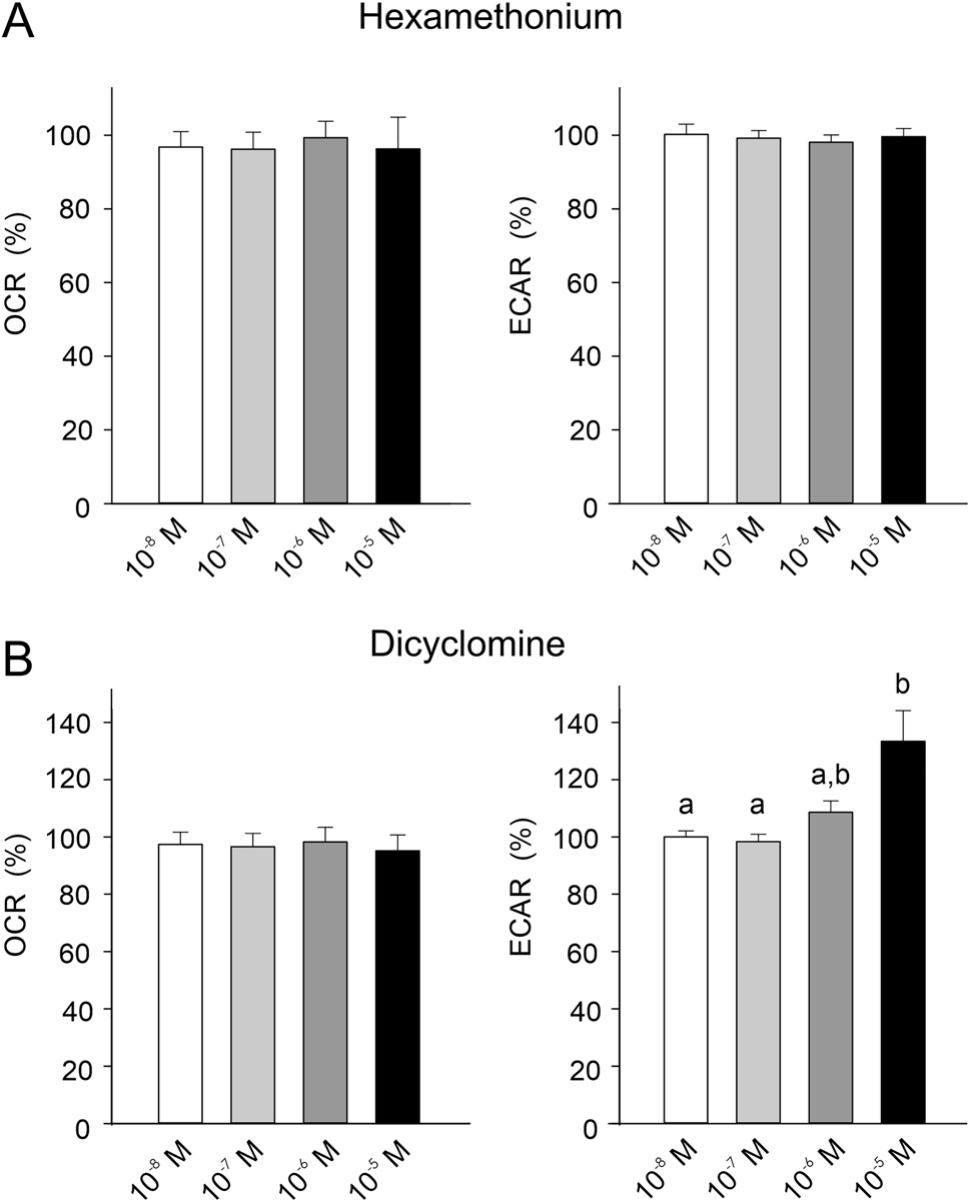
**A** The influence of an inhibition of nicotinic acetylcholine receptor by increasing concentrations of hexamethonium on OCR and ECAR. Neither OCR nor ECAR were significantly affected by inhibition of the nicotinic receptor. **B** The influence of an inhibition of muscarinic acetylcholine receptor by application of dicyclomine on OCR and ECAR. OCR was not affected by inhibition of the muscarinic receptor, but ECAR increased with higher concentrations of dicyclomine. Different small letters indicate differences between treatments *N* = 5; *p* < 0.05

**Fig. 8:**
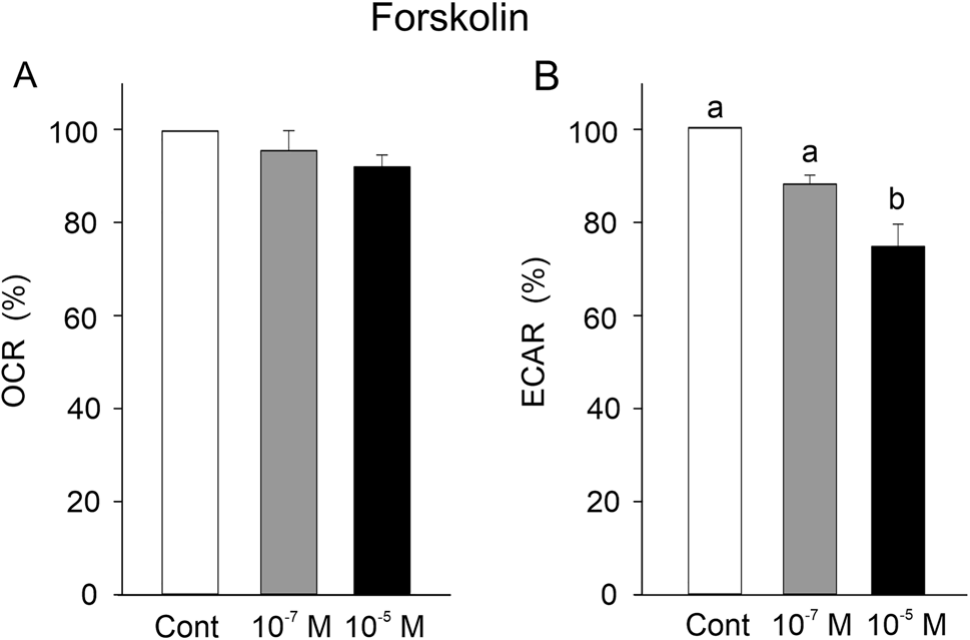
The influence of a stimulation of adenylate cyclase by increasing concentrations of forskolin on OCR (**A**) and ECAR (**B**). Forskolin had no effect on OCR, but ECAR was reduced at higher concentrations of forskolin. Different small letters indicate differences between treatments; *N* = 5; *p* < 0.05

To assess the possible role of acetylcholine and acetylcholine receptors, we incubated the cells with acetylcholine and specific acetylcholine receptor inhibitors. Incubation with acetylcholine up to a concentration of 10^–4^ mol l^−1^ did not significantly affect acid secretion (data not shown). Similarly, hexamethonium, an inhibitor of nicotinic acetylcholine receptors, had no effect (Fig. 7A), but inhibition of the muscarinic receptor by application of dicyclomine up to a concentration of 10^–5^ mol l^−1^ resulted in a concentration-dependent increase in acid secretion (Fig. 7B). None of these treatments had any effect on oxygen consumption of the cells (Fig. 7A, B). Forskolin, an adenylate cyclase activator, caused a dose-dependent decrease in acid secretion, but it had no significant effect on oxygen consumption of the cells (Fig. 8).

The adrenergic agonist dopamine was tested up to 10^–5^ mmol l^−1^, but there was no significant effect on either oxygen uptake or on acid secretion. Similarly, neither serotonin (10^–7^–10^–6^ mol l^−1^), nor gamma-aminobutyric acid (GABA; 10^–7^–10^–5^ mol l^−1^) application had any significant effect on ECAR or OCR (data not shown).

## Discussion

Gas gland cell metabolism is mainly fueled by blood glucose, glycogen stores appear to be of limited value for the acid production (Fänge 1983; Pelster 1995b). Based on glucose uptake and the expression of glucose transport proteins, it was suggested that glucose uptake via the GLUT-1 protein would determine the rate of glucose utilization in cod gas gland cells (Hall et al. 2014; Clow et al. 2016). It was expected that in silver eels swimbladder function would be improved as compared to yellow eels because during their spawning migration to the Sargasso Sea silver eels experience significantly larger changes in hydrostatic pressure as compared to yellow eels, dwelling in the European freshwater system, although the actual role of the swimbladder during the migration remains enigmatic (Pelster 2015). Following the hypothesis that glucose uptake would determine the rate of glucose utilization and thus the rate of acid secretion silver eels should have an increased capacity for glucose uptake. Transcript levels of the members of the SLC2 (GLUT transporter) and SLC5 (SGLT transporter) families of glucose transporters measured in gas gland cells of European yellow and silver eels, however, were not different between yellow and silver eels, and even infection of the swimbladder with the nematode *Anguillicola crassus*, which significantly impairs swimbladder function (Würtz et al. 1996), hardly affected the transcript levels. It therefore was concluded that changes in transcript levels would not support the hypothesis that glucose uptake is the rate-limiting step in lactate metabolism in eels (Schneebauer et al. 2018). This does not preclude the possibility, however, that the number of transport proteins may be affected by changes in translational efficiency or even at the post-translational level. Our measurements of the rate of acid secretion with increasing glucose availability now revealed that in eel gas gland cells acid secretion reached a maximum at a glucose concentration of only 1.5 mmol l^−1^, which is far below the level of glucose measured in arterial and venous blood samples collected from the dorsal aorta and from swimbladder veins of the European eel, which were in the range of 6–8 mmol l^−1^ (Pelster and Scheid 1993). We therefore conclude that the available glucose concentration is not rate-limiting for the production and secretion of acid in European eel gas gland cells.

Our measurements revealed some metabolic activity in the absence of glucose and also after complete inhibition of glucose turn-over by application of IAA, 6-AN and 2-DG. The incubation medium included 2 mmol l^−1^ glutamine. Previous analysis of the transcriptome consistently revealed presence of glutaminase transcripts in eel gas gland cells (Pelster et al. 2016; Schneebauer et al. 2017, 2020). It therefore appears quite possible that in the absence of glucose, gas gland cells used glutamine as a fuel, which would explain the low metabolic activity recorded under these conditions.

The largest reduction in acid secretion was achieved by inhibition of the glycolytic pathway using iodo-acetate (Fig. 3), confirming that lactic acid is the main end product of eel gas gland cell glucose metabolism (Pelster 1995a; Pelster and Scheid 1993). The production of lactate in the presence of oxygen and of functioning mitochondria is known as the Warburg effect (Racker 1972; Liberti and Locasale 2016), a phenomenon well described for cancer cells (Zheng 2012; Fadaka et al. 2017). The function of the Warburg effect has been connected to biosynthesis and cell proliferation, rapid ATP production, or cell signaling (Liberti and Locasale 2016). Neither of these explanations appears applicable for gas gland cells. Excessive proliferation is not expected, maintenance, or renewal of the extracellular matrix to retain or improve the gas-tightness of swimbladder tissue, may only occasionally require synthesis of lipids. In gas gland cells, production of lactic acid clearly is required for the production of acid, essential for acidification of the blood to switch on the Root effect. Acidification of the blood reduces the oxygen carrying capacity of hemoglobin. Partial deoxygenation of hemoglobin results in an increase in oxygen partial pressure, the so-called single concentrating effect, necessary for subsequent countercurrent concentration and the generation of hyperbaric oxygen tensions in the rete mirabile of the swimbladder (Kuhn et al. 1963; Pelster 2001). High gas partial pressures are required to fill the swimbladder by diffusional transport of gases, and oxygen and CO_2_ have been shown to be the main gases secreted into the eel swimbladder (Kobayashi et al. 1990).

In gas gland cells, glucose can also be metabolized in the pentose phosphate shunt (Pelster et al. 1994; Walsh and Milligan 1993). Inhibition of 6-phosphogluconate dehydrogenase caused a reduction in acid secretion, demonstrating that CO_2_ production in the pentose phosphate shunt significantly contributed to acid production. Compared to control cells, ECAR was reduced by about 30% in cells incubated with 6-AN (Fig. 3A), pre-incubation of cells with 6-AN even caused a slightly higher reduction in acid secretion (Fig. 4A).

Back-diffusion of CO_2_ in the rete mirabile has been shown to enhance the capacity of the rete mirabile to generate high oxygen partial pressures (Kobayashi et al. 1990). Our results now show that CO_2_ production in the pentose phosphate shunt also supported the initial acidification of blood during passage of the gas gland cells, underlining the crucial role of CO_2_ in swimbladder function. In a recent study, we could demonstrate the presence of aquaporin 1 in membranes of gas gland cells and also in membranes of endothelial cells of the swimbladder vasculature (Drechsel et al. 2022). Aquaporin 1 is known to be permeable to CO_2_ (Chen et al. 2010; Talbot et al. 2015; Endeward et al. 2017). We therefore hypothesized that aquaporin facilitates CO_2_ diffusion in the swimbladder, i.e., diffusion of CO_2_ from gas gland cells into the swimbladder lumen filling the swimbladder, and diffusion of CO_2_ into the blood, supporting acidification and thus switching on the Root effect.

The pentose phosphate shunt also generates NADPH, required, for example, for macromolecular biosynthesis and reactive oxygen species (ROS) or xenobiotic detoxification. Our results revealed that inhibition of the pentose phosphate shunt did not result in a more oxidized state of gas gland cells. While the concentration of reduced NADPH remained stable (except for the highest concentration of 6-AN), the concentration of the oxidized form NADP^+^ was reduced in presence of 6-AN. This suggested that the oxidized molecule was degraded to keep a reduced state of the cell, necessary to combat oxidative stress (Tao et al. 2017), and eel swimbladder tissue has been shown to have a high capacity for ROS degradation (Schneebauer et al. 2016).

Although anaerobic glycolysis is dominating in gas gland cells, some oxygen consumption has been observed, but attempts to stimulate oxygen uptake of swimbladder tissue were not successful (Ball et al. 1955; Ewart and Driedzic 1990). Our results revealed that oxygen uptake of eel gas gland cells was highest in the absence of glucose, and decreased with increasing glucose concentrations up to 1.5 mmol l^−1^. As already discussed glutamine, present in the incubation medium, may have been used as substrate for mitochondrial ATP production, required to fuel ATP consuming reactions in gas gland cells. But with sufficient glucose availability glycolytic ATP production adds to cellular ATP supply, and flux through the respiratory chain was reduced.

Assuming that oxidative phosphorylation results in an ATP production of about 32 mmol per mmol of glucose consumed, the recorded oxygen uptake of about 18–20 pmol min^−1^ (Fig. 3) would result in an aerobic ATP production of about 95–106 pmol min^−1^. Calculation of anaerobic ATP production based on ECAR can only be a rough estimate because lactic acid release may not be instantaneous and some protons may be buffered by intracellular buffer substances. The buffer capacity of the medium is extremely low and probably can be ignored. Medium pH was set to 7.4 (i.e., free proton concentration (H^+^) equaled 10^–7.4^ mol l^−1^ (= 39.811 nmol l^−1^). Ignoring possible intracellular buffering, a change in ECAR of about 20–28 mpH min^−1^ (Fig. 3, control and 6-AN) would cause a change in free proton concentration in the range of 700–1.100 pmol min^−1^. One mmol of glucose fermentation results in the production of 2 mmol lactic acid (lactate + H^+^) and 2 mmol ATP. Thus, the change in free proton concentration matches with ATP production, indicating that anaerobic ATP production in these gas gland cell preparations exceeded aerobic ATP production by a factor of 8–10. Including mitochondria-independent respiration (see below) in this calculation, the factor would even be larger.

This may explain our observation that an inhibition of the glycolytic pathway had no significant effect on oxygen consumption. If the inhibition was not complete, the remaining ATP production may have been sufficient so that an activation of aerobic metabolism was not required. The mechanisms causing a reduction in oxygen consumption with increasing glucose concentrations even in the presence of hyperoxic conditions are not yet understood. Interestingly, hypoxia even resulted in down-regulation of glucose uptake and lactate formation in gas gland tissue (Pelster and Scheid 1993).

Inhibition of mitochondrial respiration significantly reduced oxygen consumption, but in presence of rotenone and antimycin A, a fraction of mitochondria-independent respiration remained (Fig. 5). Most cells are known for non-mitochondrial oxygen consumption, caused by NADPH oxidases, desaturases and some detoxification enzymes (Brand and Nicholls 2011; Rose et al. 2014a, b). In most cells, mitochondria-independent respiration appears to contribute about 10% to total oxygen consumption (Brand and Nicholls 2011), but in gas gland cells, it was somewhat elevated with a value near 25%. Mitochondrial dysfunction has been reported to result in elevated non-mitochondrial respiration (Rose et al. 2014a, b), suggesting that in gas gland cells, the respiratory coupling and mitochondrial function may be not ideal. It also appears possible that the high ROS defense capacity of gas gland tissue (Pelster and Wood 2018; Schneebauer et al. 2016) contributes to non-mitochondrial respiration.

In the European eel, the rate of gas secretion is quite variable, depending on blood flow through the swimbladder, but also on the rate of glucose turn-over and lactate production (Pelster and Scheid 1992, 1993), suggesting that the rate of acid production and/or secretion can be regulated. In cod, *Gadus morhua*, high concentrations of acetylcholine have been shown to stimulate lactate formation to some extent (Ewart and Driedzic 1990), but we could not detect any increase in acid secretion with application of acetylcholine in our experiments with eel gas gland cells. While inhibition of the nicotinic acetylcholine receptor had no effect, inhibition of the muscarinic receptor caused a slight but significant increase in acid secretion. It thus cannot be excluded that muscarinic cholinergic stimuli may enhance acid production and secretion.

Forskolin caused a dose-dependent decrease in acid secretion, as previously observed in eel gas gland cells (Pelster and Pott 1996). Therefore, a cAMP-dependent second messenger pathway is involved in control of gas gland cell metabolism. Isoproterenol also induced a decrease in acid secretion, suggesting that ß-adrenergic signaling is involved (Pelster and Pott 1996). Epinephrine (Pelster and Pott 1996) and dopamine (this study), however, did not modify the rate of acid secretion. None of the other agonists tested in the present study had any effect on the rate of acid secretion. Noteworthy, none of the tested compounds did have any effect on oxygen uptake of gas gland cells, indicating that control of acid production is not in any way connected to the control of oxygen consumption. In fact, oxygen consumption decreased with initial glucose supply to the cells, but with glucose concentrations above 1.0–1.5 mmol l^−1^ none of our treatments had any effect on oxygen consumption.

The results of our study thus revealed that lactic acid production is the main source of acid production and acid secretion in gas gland cells of the European eel, but the pentose phosphate shunt also significantly contributes to acid production. Given the low concentration of glucose required for maximal stimulation of acid secretion, glucose uptake can hardly be the rate-limiting step for acid secretion in eel gas gland cells. Oxygen consumption of gas gland cells is reduced with increasing levels of lactate production. In the presence of glucose, oxygen consumption could not be stimulated by inhibition of glycolysis or of the pentose phosphate pathway, indicating an independent regulation of mitochondrial oxidative phosphorylation and of acid production.
